# Computational Tactics for Precision Cancer Network Biology

**DOI:** 10.3390/ijms232214398

**Published:** 2022-11-19

**Authors:** Heewon Park, Satoru Miyano

**Affiliations:** 1 M&D Data Science Center, Tokyo Medical and Dental University, 1-5-45 Yushima, Bunkyo-ku, Tokyo 113-8510, Japan; 2 Human Genome Center, Institute of Medical Science, University of Tokyo, 4-6-1 Shirokane-dai, Minato-ku, Tokyo 108-8639, Japan

**Keywords:** gene regulatory network, computational cancer biology, precision medicine, oxaliplatin and capecitabine (XELOX)

## Abstract

Network biology has garnered tremendous attention in understanding complex systems of cancer, because the mechanisms underlying cancer involve the perturbations in the specific function of molecular networks, rather than a disorder of a single gene. In this article, we review the various computational tactics for gene regulatory network analysis, focused especially on personalized anti-cancer therapy. This paper covers three major topics: (1) cell line’s (or patient’s) cancer characteristics specific gene regulatory network estimation, which enables us to reveal molecular interplays under varying conditions of cancer characteristics of cell lines (or patient); (2) computational approaches to interpret the multitudinous and massive networks; (3) network-based application to uncover molecular mechanisms of cancer and related marker identification. We expect that this review will help readers understand personalized computational network biology that plays a significant role in precision cancer medicine.

## 1. Introduction

Gene regulatory network describes functional interactions between genes, where the network is presented by a graph whose nodes present the genes, and the edges between nodes represent the regulatory interactions between genes [1,2]. Heterogeneous gene regulatory system is a useful tool to analyse and visualize biological activities and is crucial to understanding complex biological processes of cancer, because the molecular mechanisms underlying diseases reflect the perturbations in a specific function of molecules in the complex cellular network, rather than a consequence of an abnormality in a single gene [3].

The molecular interplays between genes involved in cellular processes and pathways can be represented by statistical and mathematical models. The computational strategies to estimate large-scale gene networks from gene expression levels have drawn a large amount of attention. The Gaussian graphical model (GGM), that is the probability model, has often been used to infer the conditional dependence structure of a set of genes. The GGM represents which genes (variables) predict one another and allows for sparse modeling of covariance structures, highlighting potential causal relationships between genes [4]. The Bayesian network (BN) is also a probabilistic graphical model describing a directed acyclic graph. BN has been used to uncover cancer mechanisms, i.e., unique cancer molecular mechanisms of clone cancer [5], causal networks of breast metastasis to bone, brain, or lung [6], assessing the risk of breast cancer [7], etc. Boolean networks are discrete models and one of the most widely used techniques to estimate the gene regulatory system. In the model, gene expression levels are discretized, and each gene takes on two values, i.e., if the gene expression is above a threshold value, then 1, otherwise 0, and the interactions between genes are described by standard logic (Boolean) functions [8]. Various cancer research has been based on Boolean networks for cancer drug discovery [9], identifying lung cancer diagnostic and prognostic biomarkers [10], uncovering the mechanisms of tumorigenesis and possible treatment responses of prostate cancer [11], etc. Additionally, various computational models and strategies (e.g., differential equation-based Model, artificial neural network (ANN) approaches, correlation network, information theory, etc.) have been developed and applied to cancer research. Furthermore, the effectiveness of the networks-based analysis has been proven in various fields of research, e.g., cancer prediction, drug combinations identification, and protein-protein interaction [12,13,14].

Although many computational tactics for gene regulatory network estimation have been developed and numerous studies have been conducted to uncover cancer mechanisms based on the estimated gene networks, the existing studies were conducted by an averaged gene network for all cell lines. Thus, we cannot effectively identify crucial information for precision cancer medicine.

In this article, we reviewed the computational strategies for the cell line’s (or patient’s) cancer characteristics specific gene network analysis. Especially, we reviewed machine learning approaches for varying coefficient models, where the varying coefficients describe the strength of the interaction between genes for a specific characteristic of each cell line. That is, the model enables us to construct a gene regulatory network for a specific status related to cancer of the cell line. The cell line characteristic specific gene networks estimation provides hundreds of networks for hundreds of cell lines, where each network is given as a matrix form with about 20,000 columns for target genes, 2000 rows for regulator genes, and the elements of the matrix indicate the strength of interaction between the regulator and target genes. The analysis and interpretation of the multiple and massive networks are quite difficult tasks and have remained a serious challenge in computational biology. In this article, we also review some computational tactics for comprehensive analysis and interpretation of the large-scale networks.

The remainder of this paper is organized as follows. In the gene regulatory network estimation section, the regression framework to gene regulatory network estimation is represented. The computation tactics estimate the cell line characteristic specific gene regulatory network in the sample-specific gene network estimation section. In the section of gene network analysis in multi-dimensional cell line space, the machine learning and Artificial Intelligence (AI) approaches to comprehensive analysis of the estimated multiple and massive gene regulatory network are represented. In the Applications section, the application result of the reviewed computation strategies for network-based anti-cancer drug prediction and related markers identification is introduced. Conclusions are provided in the Discussion section.

## 2. Gene Regulatory Network Estimation

Suppose $X={\left(x_{1},\ldots ,x_{n}\right)}^{T}\in R^{n\times p}$ is an n×p data matrix describing the expression of *p* regulators that may control the transcription of $\ell^{th}$ target gene $y_{\ell }\in R^{n},\ell =1,\ldots ,q$. Consider the linear regression model,

(1) $$\begin{array}{c} y_{\ell }=\sum_{j=1}^{p}\beta_{j\ell }x_{j}+\epsilon_{\ell },\;\ell =1,\ldots ,q, \end{array}$$

where $\beta_{j\ell }$ describes the effect of the $j^{th}$ regulator gene on the $\ell^{th}$ target gene, and $\varepsilon_{\ell }$ is a random error vector $\varepsilon_{\ell }={\left(\varepsilon_{\ell 1},\ldots ,\varepsilon_{\ell n}\right)}^{T}$ that is assumed to be independently and identically distributed with mean 0 and variance $\sigma^{2}$. To estimate the gene regulatory network, the following $L_{1}$-type regularization methods were used successfully,

(2) $$\begin{array}{c} L\left(\beta_{\ell }\right)=\underset{\beta_{\ell }}{\arg\;\min}\left\{\frac{1}{2}\sum_{i=1}^{n}{\left(y_{i\ell }-\sum_{j=1}^{p}\beta_{j\ell }x_{ij}\right)}^{2}+P\left(\beta_{\ell }\right)\right\}, \end{array}$$

where

- ridge [15]: $P\left(\beta_{\ell }\right)=\lambda \sum_{j=1}^{p}\beta_{j\ell }^{2}$
- lasso [16]: $P\left(\beta_{\ell }\right)=\lambda \sum_{j=1}^{p}\left|\beta_{j\ell }\right|$
- elastic net [17]: $P\left(\beta_{\ell }\right)=\lambda \sum_{j=1}^{p}\{\gamma \beta_{j\ell }^{2}+\left(1-\gamma \right)|\beta_{j\ell }\left|\right\}$
- etc.

and λ,γ>0 are the regularization parameters, where λ controls model complexity, and γ is a mixing parameter between the lasso and ridge penalties. The $L_{1}$-type regularization methods enable us to simultaneously identify crucial regulators and estimate their effect on a target gene. In particular, the methods effectively perform analysis of the high dimensional genomic alterations dataset.

Although the methods successfully perform edge selection and network estimation, the approaches provide an averaged network for all *n* cell-lines. Thus, we cannot estimate cell line (or patient) characteristic-specific models (i.e., molecular interplay). In other words, the methods are not enough to extract useful information for precision medicine.

## 3. Sample-Specific Gene Network Estimation

To effectively extract crucial information for precision medicine, cell line (or patient) characteristic-specific identification is a crucial issue. We reviewed computational approaches for cell-line characteristic-specific modelling, especially cell line characteristic-specific gene regulatory network estimation. The following varying coefficient model was used for cell-line characteristic-specific modelling [18],

(3) $$\begin{array}{c} y_{\ell }=\sum_{j=1}^{p}\beta_{j\ell }\left(m_{\alpha }\right)\cdot x_{j}+\epsilon_{\ell },\;\ell =1,\ldots ,q,\;\alpha =1,\ldots ,n, \end{array}$$

where $\beta_{j\ell }\left(m_{\alpha }\right)$ describes the effect of the $j^{th}$ regulator gene on the $\ell^{th}$ target gene in the $\alpha^{th}$ target cell line. $m_{\alpha }$ is a cancer related characteristic of the $\alpha^{th}$ cell lines, such as drug sensitivity and survival risk of cell lines. The model enables us to describe cell-line characteristic- ($M=m_{\alpha }$) specific molecular interplay between genes, i.e., $\beta_{j\ell }\left(m_{\alpha }\right)$.

### 3.1. NetworkProfiler

The varying coefficient $\beta_{j\ell }\left(m_{\alpha }\right)$ describing cell-line characteristic-specific strength of the relationship between the $j^{th}$ regulator and the $\ell^{th}$ target genes in the $\alpha^{th}$ cell line can be estimated by the following kernel-based $L_{1}$-type regularization method, called a NetworkProfiler [19],

(4) $$\begin{array}{c} L\left(\beta_{\ell \alpha }|b_{\ell }\right)=\frac{1}{2}\;\sum_{i=1}^{n}{\{y_{i\ell }\;-\;\sum_{j=1}^{p}\beta_{j\ell }\left(m_{\alpha }\right)x_{ij}\;\}}^{2}G\left(m_{i}\;-\;m_{\alpha }|b_{\ell }\right)\;+P\left(\beta_{\ell \alpha }\right), \end{array}$$

where

(5) $$\begin{array}{c} G\left(m_{i}-m_{\alpha }|b_{\ell }\right)=\exp\left\{\frac{-{\left(m_{i}\;-\;m_{\alpha }\right)}^{2}}{b_{\ell }}\right\}, \end{array}$$

is a Gaussian kernel function to control the weight of cell lines when modelling the $\alpha^{th}$ target cell line. The NetworkProfiler groups cell lines, according to the similarity of the specific characteristics of cell lines (i.e., modulator $m_{i}$ for i=1,…,n), and performs modelling for $\alpha^{th}$ cell lines, based only on the cell-lines in the neighbourhood around the $\alpha^{th}$ cell line. That is, the modelling for the $\alpha^{th}$ cell line is based only on the cell lines having similar modulator characteristics to with the target sample’s modulator value $m_{\alpha }$. This implies that the NetworkProfiler can estimate cell line characteristic -specific gene regulatory networks.

**Figure 1:**
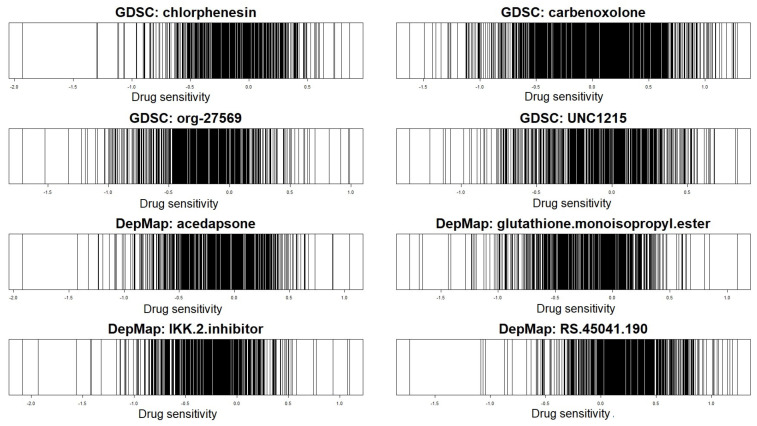
Drug sensitivities of GDSC and DepMap databases: each of the four drugs are randomly selected from GDSC and DepMap datsets.

The cancer-related characteristics of cell lines are not usually uniformly distributed. Figure 1 shows the anti-cancer drug sensitivity of cell lines, where the eight drugs are randomly selected from Genomics of Drug Sensitivity in Cancer (GDSC) and Cancer Dependency Map (DepMap) projects. As shown in Figure 1, sensitivities of some anti-cancer drugs (characteristics of cell lines: modulator) are non-uniformly distributed, i.e., there are cell lines having rare cancer characteristics.

**Figure 2:**
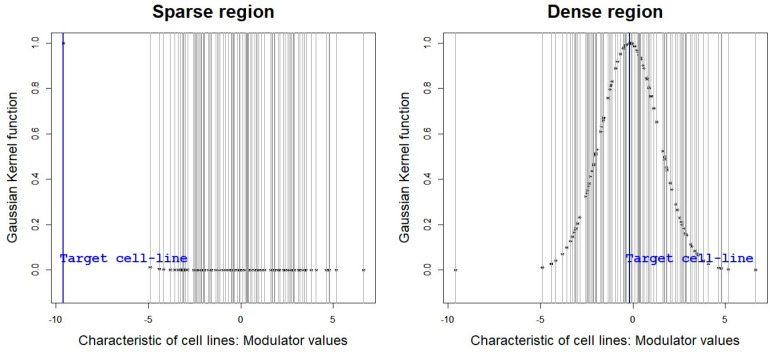
Gaussian kernel function to impose weight on cell lines, where *y*-axis and *x*-axis indicate weight and modulator values of cell lines.

**Limitation:**  The NetworkProfiler cannot perform well when the modulator is not uniformly distributed, especially when modelling the target cell line with a rare characteristic located in a sparse region of its distribution, because the method is based on the constant bandwidth ($b_{\ell }$) of Gaussian kernel function. In the NetworkProfiler, the bandwidth specifies the length-scale of the kernel function and controls the weights of cell lines. It implies that the NetworkProfiler based on the constant bandwidth performs cell line characteristic-specific modelling without consideration of the distribution of the modulator and location of the modulator value $m_{\alpha }$ of the target sample in the distribution. Thus, the NetworkProfiler imposes a small amount of weight to almost all samples for modelling a target sample in a sparse region. Figure 2 shows the values of the Gaussian kernel function (i.e., weight for cell lines) with a constant bandwidth $b_{\ell }$ for a target sample in both sparse and dense regions, where *y*-axis and *x*-axis indicate weights and modulator values of cell-lines, respectively. As shown in Figure 2, the Gaussian kernel function based on the constant bandwidth imposes the non-zero weight on only a few samples for the modelling of the target sample in a sparse region. It leads to extremely high dimensional data situations; thus, gene regulatory network estimation (i.e., edges selection and edge size estimation) cannot be appropriately performed.

### 3.2. Adaptive NetworkProfiler

To settle on the issue, Park et al. [20] developed a novel strategy, called an adaptive NetworkProfiler, based on the adaptive bandwidth of the Gaussian function. The adaptive NetworkProfiler computes the weight of cell lines by using the adaptive Gaussian kernel function, where the bandwidth is based on the *k*-nearest neighbour (KNN) rule, called an adaptive bandwidth [21]. The adaptive bandwidth for an $\alpha^{th}$ target cell line is based on the Euclidean distance between the modulator value of $\alpha^{th}$ cell line ($m_{\alpha }$) and its $k^{th}$ nearest neighbour. By using not a constant but the adaptive bandwidth based on Euclidean distance, the KNN-Gaussian kernel function has a relatively wide width of the kernel for modelling a target sample, having a rare modulator value located in the sparse region, because the $k^{th}$ nearest neighbourhood of $\alpha^{th}$ target cell line is also far from the $m_{\alpha }$. Thus, the KNN-Gaussian kernel function can overcome the drawback of the ordinary NetworkProfiler for modelling a target sample in the sparse region.

The adaptive NetworkProfiler was developed based on the adaptive kennel function, with an additional parameter incorporating dispersion of modulators (i.e., range of a modulator: r(M)) as follows,

(6) $$\begin{array}{c} L\left(\beta_{\ell \alpha }|b_{\ell }^{\mathrm{KNN}},r\left(M\right)\right)=\frac{1}{2}\;\sum_{i=1}^{n}{\left\{y_{i\ell }\;-\;\sum_{j=1}^{p}\beta_{j\ell }\left(m_{\alpha }\right)x_{ij}\;\right\}}^{2}K\left(m_{i}\;-\;m_{\alpha }|b_{\ell \alpha ,r(M)}^{\mathrm{KNN}}\right)+P\left(\beta_{\ell \alpha }\right), \end{array}$$

where

(7) $$\begin{array}{c} K\left(m_{i}-m_{\alpha }|b_{\ell \alpha }^{\mathrm{KNN}},r\left(M\right)\right)=\exp\left(\frac{-{\left(m_{i}\;-\;m_{\alpha }\right)}^{2}}{b_{\ell \alpha }^{\mathrm{KNN}}\cdot r\left(M\right)}\right), \end{array}$$

and $b_{\ell \alpha }^{\mathrm{KNN}}$ is the Euclidean distance between $m_{\alpha }$, with its $k^{th}$ nearest neighbour $m_{\alpha }^{k^{th}}$,

(8) $$\begin{array}{c} b_{\ell \alpha }^{\mathrm{KNN}}=\sqrt{{\left(m_{\alpha }-m_{\alpha }^{k^{th}}\right)}^{2}}\;\mathrm{for}\;\alpha =1,2,\ldots ,n, \end{array}$$

and r(M) is the hyperparameter, incorporating dispersion of the modulator *M*.

The adaptive bandwidth in the Gaussian kernel function with the additional parameter incorporates distribution of cell line characteristics (*M*) and location of the characteristic value ($m_{\alpha }$) in their distribution. Thus, the adaptive NetworkProfiler can overcome only a small number of samples that have non-zero weight and can effectively perform cell line characteristic-specific gene network estimation for modelling the target sample in not only dense regions but also sparse regions.

**Limitation:**  The NetworkProfiler and Adaptive NetworkProfiler construct the cancer characteristics-specific gene network based on a specific cancer characteristic. That is, the methods consider a characteristic and measure similarity of cell lines in one-dimensional cell line characteristic space based only on one characteristic. Thus, the cancer characteristic-specific gene networks estimated by the methods cannot described gene regulatory system under varying conditions of various cancer characteristics because the methods are based on a characteristic.

### 3.3. Gene Network Analysis in Multi-Dimensional Cell Line Space

In order to incorporate various cancer-related characteristics of cell lines and extract more precise cell-line specific molecular interplays, the cell line characteristic specific gene network estimation is extended to the multi-dimensional cell line space [22].

For *h* characteristics of cell line $M=\left(m_{1},\ldots ,m_{h}\right)\in R^{n\times h}$, the varying coefficient model in (3) is given as follows,

(9) $$\begin{array}{c} y_{\ell }=\sum_{j=1}^{p}\beta_{j\ell }\left(m_{\alpha }\right)\cdot x_{j}+\epsilon_{\ell },\;\ell =1,\ldots ,q,\;\alpha =1,\ldots ,n, \end{array}$$

where $m_{\alpha }=\left(m_{\alpha 1},\ldots ,m_{\alpha h}\right)$. In the *h*-dimensional cell line space, the similarity between cell lines is measured by the following multivariate Gaussian kernel function,

(10) $$\begin{array}{c} K\left(m_{i}-m_{\alpha }|H_{\ell }\right)={\left|H_{\ell }\right|}^{-1/2}\exp\left\{-\frac{1}{2}{\left(m_{i}-m_{\alpha }\right)}^{T}H_{\ell }^{-1}\left(m_{i}\;-\;m_{\alpha }\right)\right\} \end{array}$$

where $H_{\ell }$ is the bandwidth matrix (e.g., covariance matrix). Then, the multi-dimensional cell line characteristic specific gene network is estimated by the following multivariate kernel-based $L_{1}$-type regularization method,

(11) $$\begin{array}{c} L\left(\beta_{\ell \alpha }|H_{\ell }\right)=\frac{1}{2}\;\sum_{i=1}^{n}{\left\{y_{i\ell }\;-\;\sum_{j=1}^{p}\beta_{j\ell }\left(m_{\alpha }\right)x_{ij}\;\right\}}^{2}K\left(m_{i}-m_{\alpha }|H_{\ell }\right)\;+P\left(\beta_{\ell \alpha }\right). \end{array}$$

The multi-dimensional cell line characteristic specific analysis enables us to extract more precise characterization of cell-lines, and thus we can effectively estimate precision cancer gene regulatory networks.

**Limitation:**  The precision cancer gene networks estimation provides hundreds of matrices with more than 2000 rows for regulator genes and more than 10,000 columns for target genes. Although various computational tactics have been developed and successfully applied to gene regulatory network estimation, the interpretation of the large-scale gene networks remains a challenge. The existing studies on the cell line characteristic-specific gene networks focused only on the known markers and then interpreted the massive networks based on the neighbourhoods of the known markers, i.e., only narrow interpretation was performed. However, comprehensive analysis of the multiple massive networks is essential to understand the complex mechanism of cancer. The interpretation of the multi-layer massive network was the bottle network of the existing studies on the precision cancer gene networks analysis.

## 4. Interpretation of the Multi-Layer Massive Networks

In this section, we review computational strategies to interpret the multiple and massive gene regulatory networks.

### 4.1. Network Constrained Sparse Common Component Analysis (NetSCCA)

Park et al. [22] considered common structure identification of the multiple matrix datasets to interpret multilayer massive networks. The cell line specific gene regulatory system can be described by the following regulatory effect of the $j^{th}$ regulator gene on the $\ell^{th}$ target gene in the $\alpha^{th}$ cell line [19,22],

(12) $$\begin{array}{c} r_{\alpha lj}={\hat{\beta }}_{\ell j}\left(m_{\alpha }\right)\cdot x_{\alpha j},\;\mathrm{for}\;j=1,\ldots ,p, \end{array}$$

where $x_{\alpha j}$ is an expression level value of the $j^{th}$ gene in the $\alpha^{th}$ cell line. For the $\ell^{th}$ target gene, a matrix for the regulatory effect of *p* regulators is given as $R_{\ell }={\left(r_{1\ell },\ldots ,r_{n\ell }\right)}^{T}\in R^{n\times p}$, where $r_{\alpha \ell }={\left(r_{\alpha \ell 1},\ldots ,r_{\alpha \ell p}\right)}^{T}$.

To interpret the large-scale gene regulatory networks and identify crucial biomarkers that play a key role in cancer-related mechanism of interest, the network-constrained sparse common component analysis (NetSCCA) was developed. The crucial common component of multiple datasets ($R_{\ell },\ell =1,\ldots ,q$) can be estimated by [23],

(13) $$\begin{array}{c} \underset{A}{\arg\;\min}\{\sum_{\ell =1}^{q}∥R_{\ell }-R_{\ell }AA^{T}{∥}_{F}^{2}\},\\ \mathrm{subject}\mathrm{to}\;A^{T}A=I_{K}. \end{array}$$

As show in (13), the common component analysis of *q* datasets can be considered as a principal component analysis (PCA) of *q* datasets. That is, if there is only one dataset $R_{1}$, then the model becomes a standard PCA. Wang et al. [23] showed that the common loading matrix A in (13) can be optimized as the solution to the following problem,

(14) $$\begin{array}{c} \underset{A}{\arg\;\max}\mathrm{Tr}\left(A^{T}GA\right),\\ \mathrm{subject}\mathrm{to}\;A^{T}A=I_{K}. \end{array}$$

where $G=\sum_{\ell =1}^{q}R_{\ell }^{T}R_{\ell }$. It implies that the common loading matrix A can be estimated by the standard PCA problem.

(15) $$\begin{array}{c} \underset{A}{\arg\;\min}\{∥Q-QAA^{T}{∥}_{F}^{2}\},\\ \mathrm{subject}\mathrm{to}\;A^{T}A=I_{K}, \end{array}$$

where Q is the square root of G, i.e., $Q^{T}Q=G$. The common component analysis enables us to estimate the common subspace of the multiple massive networks (i.e., $R_{\ell },\ell =1,\ldots ,q$), and extract the crucial common component of the datasets.

The common component estimation in (13) provides a fully dense projection matrix A. That is, the common component is estimated by a linear combination of all features. It not only leads to difficult to interpret estimated common components but also erroneous estimation results, because the common component analysis is based on crucial and noisy features. To settle the issue, a sparse learning-based strategy was proposed and developed to achieve better biological interpretability, called a NetSCCA [22]. The NetSCCA estimates the projection matrix A based only on crucial features without disturbance of noisy features by using sparse learning and incorporates network biology knowledge that the genes with similar molecular interactions may have similar biological function in the common component estimation.

The NetSCCA measures the similarity between genes on networks by using the following jaccard similarity [24]:

(16) $$\begin{array}{c} W_{j,s}=\frac{|N_{j}\cap N_{s}|}{|N_{j}\cup N_{s}|} \end{array}$$

where $N_{j}$ is the set of nodes that are directly connected to the $j^{th}$ gene via an edge in at least one cell line. Then, the similarity between genes $W_{j,s}$ is incorporated into the sparse common loading matrix (A) estimation as follows,

(17) $$\begin{array}{c} \underset{\Theta ,A}{\arg\;\min}\{\sum_{\ell =1}^{q}∥Q-Q\Theta A^{T}{∥}_{F}^{2}\}+\lambda_{1}\sum_{k=1}^{K}{∥\theta_{k}∥}_{1}+\lambda_{2}\sum_{k=1}^{K}\sum_{j<s}{\left(\theta_{j,k}-\theta_{s,k}\right)}^{2}W_{j,s}, \end{array}$$

where $\theta_{k}\propto A_{k}$ is the *p*-dimensional vector and $\Theta =(\theta_{1},\ldots ,\theta_{k})$. The last term (penalty term) of (17) enables us to locally smooth the coefficients and encourage the simultaneous selection of related genes. In other words, a large amount of weight is imposed on the coefficients of the two genes with many common interactions, and it encourages similarity in their coefficients of common structure estimation. Thus, the NetSCCA can provide biologically interpretable results of the common component analysis of the multiple networks.

The NetSCCA algorithm is given in Algorithm 1.
**Algorithm 1** NetSCCA: Network constrained sparse common component analysis.1: Compute jaccard similarity: W.2: For *q* target genes, compute the regulator effect matrices as $R_{\ell }$ for ℓ=1,…,q and    $G=\sum_{\ell =1}^{q}R_{\ell }^{T}R_{\ell }$.3: For the square root of G (i.e., $Q^{T}Q=G$), compute sparse common loadings of *q*    regulator effect matrices $R_{\ell },\ell =1,\ldots ,q$.        3.1: Start A at $V=[V_{1},V_{2},\ldots ,V_{K}]$, which is the loading matrix from ordinary PCA of Q.        3.2: Given a fixed $A=[a_{1},a_{2},\ldots ,a_{K}]$, solving the following problem,              $\begin{array}{c} {\hat{\theta }}_{k}\underset{\theta_{k}}{\arg\;\min}\{∥z_{k}-Q\theta_{k}{∥}^{2}\}+\lambda_{1}{∥\theta_{k}∥}_{1}+\lambda_{2}\sum_{j<s}{\left(\theta_{j,k}-\theta_{s,k}\right)}^{2}W_{j,s}\;k=1,2,\ldots ,K, \end{array}$        where $z_{k}=Qa_{k}$. Update $\hat{\Theta }=\left[{\hat{\theta }}_{1},{\hat{\theta }}_{2},\ldots ,{\hat{\theta }}_{K}\right]$.        3.3: For a fixed $\hat{\Theta }$, perform the singular value decomposition of $Q^{T}Q\hat{\Theta }=U\Gamma V^{T}$ and
        update $\hat{A}=UV^{T}$ (see Zou et al. [25]).        3.4: Repeat Steps 3.2–3.3, until convergence.4: Sparse common loading is given by $\frac{{\hat{\theta }}_{k}}{∥{\hat{\theta }}_{k}∥}$ for k=1,…,K.

**Limitation:**  As pointed out by existing studies on network-based regularization [26,27], the network-constrained regularisation cannot perform well when the connected genes have opposite signs of coefficients. The limitation of the NetSCCA can be overcome by use of the advanced network-constrained regularization methods that incorporate signs of the regression coefficients [27].

### 4.2. Explainable AI for Gene Network-Based Prediction (Xprediction)

In this section, we review an explainable AI approach for the network-based prediction, called Xprediction [28]. Although the machine learning-based AI approaches provide effective prediction results, most of the existing approaches were developed focusing only on mathematical/statistical accuracy. Thus, the existing AI methodologies cannot explain their decision rules (i.e., the existing AI cannot explain how and why a decision has been made, causing the black-box problem). However, the interpretability and explainability are essential for use of AI strategies in various fields of research, especially medical science.

Xprediction achieves not only prediction accuracy but also interpretability of deep learning-based AI. The method is based on the widely used machine learning and deep learning approaches, e.g., the kernel support vector machine, random forest and deep neural network for prediction models, and describes the cruciality of input on output by comparison with the results of the model without the input. That is, Xprediction constructs a model by removing a feature (i.e., by removing a molecular interaction between $\ell^{th}$ target and $j^{th}$ regulator genes) individually and performing a prediction, and the prediction is iterated based on the randomly constructed cross-validation datasets. The cruciality of each molecular interaction was measured by comparing with the prediction accuracy based on all molecular interactions Acc$\left(\hat{y}\right)$.

The significance of each molecular interaction is computed by the *t*-test between prediction accuracies between models with and without the edge (i.e., interaction). Let *N* be a number of iterations for computing prediction accuracy from the randomly constructed cross-validation dataset, then $\bar{\mathrm{Acc}(\hat{y})}$ and $s_{\hat{y}}$ are mean and standard deviation of the prediction of accuracies in *N* iterations, respectively. In the model without (l,j) interaction, corresponding notations are given $N^{\left(l,j\right)}$, $\bar{\mathrm{Acc}({\hat{y}}^{\left(l,j\right)})}$ and $s_{\left({\hat{y}}^{\left(l,j\right)}\right)}$, respectively. We performed the following *t*-test,

(18) $$\begin{array}{c} T_{\ell j}=\frac{\bar{\mathrm{Acc}(\hat{y})}-\bar{\mathrm{Acc}({\hat{y}}^{\left(l,j\right)})}}{s_{p}\sqrt{\frac{1}{N}+\frac{1}{N^{\left(l,j\right)}}}} \end{array}$$

where $s_{p}=\sqrt{\frac{s_{\hat{y}}\left(N-1\right)+s_{{\hat{y}}^{\left(l,j\right)}}\left(N^{\left(l,j\right)}-1\right)}{N+N^{\left(l,j\right)}-2}}$. Then, the cruciality of ${\left(l,j\right)}^{th}$ interaction $I_{\ell j}$) on the prediction result was measured by the *p* value of the *t*-test. The algorithm of Xprediction is given in Algorithm 2.
**Algorithm 2** Xprediction: explainable prediction.1:Construct prediction models based on the kernel support vector machine (kSVM),
Random Forest (RF), and Neural Network (NN).2:Compute prediction accuracies based on *k*-fold cross-validation (CV). The average of
the prediction accuracies of *k* validation sets was given as: Acc$\left(\hat{y}\right)$.3:Step 2 is iterated *N* times for randomly constructed *k*-fold CV datasets.4:If l≤q, then5:   If j≤p, then6:      Delete (l,j) elements from regulatory effect matrices: $R_{\ell },\ell =1,\ldots ,q$7:      Compuate prediction accuracy of the model without (l,j) elements: Acc$\left({\hat{y}}^{\left(l,j\right)}\right)$.8:      Step 7 is iterated $N^{\left(l,j\right)}$ times for randomly constructed *k*-fold CV datasets.9:Perform *t*-test between Acc$\left(\hat{y}\right)$ and Acc$\left({\hat{y}}^{\left(l,j\right)}\right)$ obtained from *N* and $N^{\left(l,j\right)}$ iterations
and compute *p* value.10:Cruciality of molecular interplays for AI-based prediction results are measured by on
*p* value of the *t*-test.

**Limitation:**  The Xprediction constructs (q×p)+1 prediction models, because prediction accuracies of the model based on the regulatory effect without the (i,j) element should be compared with the model based on the regulatory effect with all elements. This leads to a great amount of computation. The computational complexity is one of limitations of the Xprediction.

## 5. Applications

In this section, we introduce an application of the introduced computational strategy to precision cancer network analysis. We consider the application of the explainable AI, Xprediction, to identify anti-cancer drug markers. The drug sensitivity data (i.e., primary-screen-replicate-collapsed-logfold-change) and RNA-expression levels of genes are obtained from the CCLE dataset (https://depmap.org/portal/, accessed on 4 August 2022). For expression levels of genes, we extracted 1922 genes that had the highest 10% variances in cell lines. We focused on anti-cancer drugs, capecitabine, and oxaliplatin, which are used in a chemotherapy combination known as XELOX or CAPEOX. The XELOX is used to colorectal and gastric cancer [29,30,31].

We first estimated capecitabine’s sensitivity specific gene networks by using the NetworkProfiler. We then defined oxaliplatin sensitive and resistant cell lines based on fifth (5P) and ninety-fifth (95P) percentiles of the drug sensitivity (DS) values, i.e., sensitive cells: DS < 5P and resistant cells: DS > 95P. We constructed a prediction model based on the deep learning approach (i.e., deep neural network) to predict the sensitivity of the oxaliplatin. In our analysis, a two hidden layered, fully-connected feed-forward neural network was used. The ReLU activation function was used on the hidden layers, and the sigmoid function was used on the output layer. We randomly split the dataset 10-fold and evaluated the prediction accuracy based on the 10-fold cross-validation, i.e., prediction accuracy was given as an average of prediction accuracies of 10 test sets. By using Xprediction, crucial molecular interactions to explain sensitivities of the oxaliplatin were identified based on *p* value < 0.05. Table 1 shows the identified crucial interactions and corresponding *p* value.

**Figure 3:**
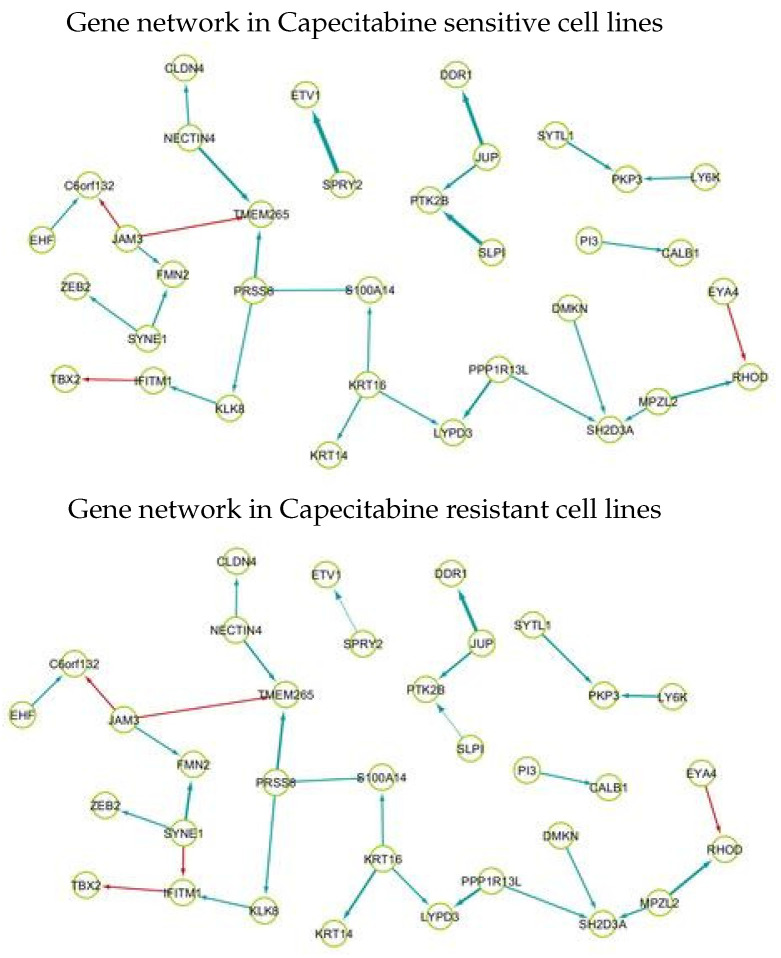
Gene regulatory networks of the crucial molecular interplays to oxaliplatin sensitivity prediction. Color of edge indicate negative (red) and positive (blue) effects of regular genes on their target genes.

Figure 3 shows gene regulatory networks consisting of the identified crucial molecular interplays to oxaliplatin sensitivity prediction, where the top and bottom indicates the networks in drug sensitive and resistance cell lines, respectively. The edge sizes represent the median of strengths of interactions between genes in drug-sensitive cell lines and -resistant cell lines, respectively.

As shown in Figure 3, drug-sensitive and-resistant cell lines show different gene regulatory systems of the identified markers. The interplay of SYNE1→ IFITM1 can be considered as a oxaliplatin-resistant specific gene regulatory system. The interplays of SPRY2→ ETV1 and SLPI → PTK1B become weaker from sensitive to resistant cell lines. It was uncovered that high expression levels of the identified drug resistant markers SYNE1 and IFITM1 are associated with poorer chemotherapy efficacy of gastric cancer and resistance to endocrine therapy and chemotherapy [32,33]. The existing studies support our results that the high activities of SYNE1 and IFITM1 are characteristics of capecitabine-resistant cell lines. On the other hand, it was demonstrated that the high expression levels of the SPRY2 are associated with chemotherapy-sensitive cell line MEK inhibitors, BRAF inhibitor-resistant cells, and ovarian cancer cells [34,35,36]. The results of the literature are consistent with our result that the activity of SPRY2 is a signature of capecitabine-sensitive cell lines. This implies that our gene network analysis results are strongly supported by existing literatures.

Table 2 shows that the genes consisted of the crucial interplays, related anti-cancer drugs, and cancer, where the column “Resistant” indicates that the gene was identified as a drug-resistant marker in existing studies. It can be seen from Table 2 that more than half of the identified genes are confirmed as a therapeutic target for not only XELOX (i.e., Oxaliplatin and Capecitabine) but also various anti-cancer drugs (e.g., 5-FU, cisplatin, Paclitaxel, etc.). Furthermore, the cancer-related mechanism of the genes has been verified in the literature. Although the mechanism of some genes has not yet been uncovered, it can be considered through our results and literatures that not just a single gene but the identified molecular interplays may be crucial to understanding the mechanism of anti-cancer drug resistance of cell lines.

We suggest though the application results of precision cancer network analysis and literature that molecular interplays between “SYNE1 and IFITM1” may lead to capecitabine resistance of cancer cell lines, while weakening the molecular regulatory interactions between “SPRY2 and ETV1” and “SLPI and PTK1B” induce capecitabine-resistance in cell lines.

## 6. Discussion

In this article, we reviewed computational tactics for precision cancer network analysis. Although many studies have been conducted to develop computational approaches to gene regulatory network analysis and the gene network-based analysis has been applied to cancer research, the existing studies focused on an averaged gene network for all cell lines. Thus, we cannot extract crucial information for precision cancer research. In this article, we have focused on cancer characteristic-specific gene networks and reviewed the computational strategies for cell line specific modelling to identified cancer characteristic-specific molecular interplays. We also reviewed the studies on analysis and interpretation of the estimated multiple and massive gene regulatory networks. Finally, we introduced the application results of the introduced computational tactics to anti-cancer drug sensitivity-specific gene network analysis. The application section described cell line’s characteristic- (drug sensitivity) specific gene regulatory network analysis. Our analysis can be easily extended to patient’s characteristic-specific gene network analysis by using expression levels and drug sensitivities summarized in each patient. We expect that the results of a cancer patient’s characteristic-specific gene network analysis provides crucial evidence for precision medicine.

Although we have reviewed some computational tactics for interpretation of multiple and massive gene networks, from cell line characteristic-specific gene network estimation to computational network biology, interpretation and analysis of the large-scale gene networks is still in its infancy. Thus, researchers in various fields of research are faced with a challenge to interpret the estimated large gene networks. Explainable machine learning and, more specifically, interpretable artificial intelligence will be a key tool to overcome this bottleneck in the near future.

## Figures and Tables

**Table 1:** Crucial molecular interplays to explain oxaliplatin sensitivity, where X→Y indicates interaction from regulator gene *X* to target gene *Y*.

Interaction	*p* Value	Interaction	*p* Value
MPZL2→SH2D3A	0.003	TP63→ITGB4	0.039
JUP→DDR1	0.008	EHF→C6orf132	0.040
DMKN→SH2D3A	0.018	PPP1R13L→LYPD3	0.040
DMKN→MPP1	0.019	IFITM1→TBX2	0.040
KRT16→S100A14	0.019	JAM3→FMN2	0.042
PRSS8→TMEM265	0.029	S100A7→KRT14	0.044
SLPI→PTK2B	0.032	KLK8→IFITM1	0.046
PI3→CALB1	0.033	EYA4→RHOD	0.046
SPRY2→ETV1	0.033	SYNE1→ZEB2	0.048
LY6K→PKP3	0.034	NECTIN4→CLDN4	0.049
SYTL1→KLK8	0.035		

**Table 2:** Identified markers and their evidence.

Genes	RG/TG	Drugs	Cancer	Resistant	Evidences
C6orf132	TG	-	-		
CALB1	TG	-	-		
CLDN4	TG	5-FU, cisplatin, Paclitaxel, cDDP	PDC, GS, CRC	*	[37,38,39,40,41]
DDR1	TG	Oxaliplatin	GS, CRC		[42,43,44]
DMKN	RG	-	CRC		[45]
EHF	RG	-	-		
ETV1	TG	oxaliplatin, 5-FU	HCC, GS, CRC	*	[46,47,48]
EYA4	RG	-	-		-
FMN2	TG	-	-		-
IFITM1	RG, TG	-	GS, CRC, EAC, GBC		[49,50,51]
ITGB4	TG	cisplatin, erlotinib, 5-Fu	LC	*	[52,53,54]
JAM3	RG	-	LIC		[55]
JUP	RG	-	-		-
KLK8	RG, TG	oxaliplatin	CRC, PC	*	[56,57]
KRT14	TG	Erlotinib	LC, BC		[58,59]
KRT16	RG	Erlotinib	BC		[59]
LY6K	RG	-	GC, BC	*	[60,61,62]
LYPD3	TG	-	AML		[63]
MPZL2	RG	-	-		-
NECTIN4	RG	5-FU, Enfortumab Vedotin	CC, BC, GC, LC	*	[64,65]
PI3	RG	-	-		-
PKP3	TG	5-FU, leucovorin, oxaliplatin	CRC		[66]
PPP1R13L	RG	5-FU	GS	*	[67]
PRSS8	RG	-	-		-
PTK2B	TG	Midostaurin, gilteritinib/defactinib, TKI	AML	*	[68]
RHOD	TG	-	-		-
S100A14	TG	-	GS		[69]
S100A7	RG	-	-		-
SH2D3A	TG	-	-		-
SLPI	RG	-	-		-
SPRY2	RG	5-FU	CC		[70]
SYNE1	RG	-	GC		[32]
SYTL1	RG	-	-		-
TBX2	TG	platinum-ased chemotherapy	OC, CRC		[71,72]
TMEM265	TG	-	-		-
TP63	RG	apatinib and capecitabine	BC		[73]
ZEB2	TG	oxaliplatin and 5-FU, cisplatin, trastuzumab	CRC, GC	*	[74,75,76]

AML: Acute myelogenous leukaemia; BC: breast cancer; CC: colon cancer; CRC: colorectal cancer; EAC: oesophageal adenocarcinoma; GBC: gallbladder cancer; GS: gastric cancer; HCC: hepatocellular carcinoma; LC: lung cancer; OC: ovarian cancer; PC: pancreatic cancer; PDC: pancreatic ductal carcinomas; cDDP: cis-diamminedichloroplatinum. The * indicates that the gene was identified as a drug resistant marker in the existing studies.

## Data Availability

The datasets used in the Application section are from the Dependency Map (DepMap) projects (https://depmap.org/portal/, accessed on 4 August 2022).
